# Embolization of medium-sized vessels with the penumbra occlusion device: evaluation of anchoring function

**DOI:** 10.1186/s42155-020-00115-4

**Published:** 2020-05-04

**Authors:** Kenichi Kato, Kazuya Kawashima, Tomohiro Suzuki, Makoto Hamano, Sohei Yoshida, Kunihiro Yoshioka

**Affiliations:** Department of Radiology, Iwate Medical University, 1-1-1 Idaidori, Shiwa-gun, Yahaba-cho, Iwate Prefecture 028-3694 Japan

**Keywords:** Embolization, Penumbra occlusion device, Interventional radiology

## Abstract

**Background:**

The penumbra occlusion device (POD) is a recently developed metallic coil with a unique anchor segment. The purpose of this study was to investigate the anchoring function of the POD for embolization of medium-sized vessels in detail.

**Materials and methods:**

We reviewed a series of cases of proximal embolization of medium-sized vessels in which the POD was used. Endovascular outcomes and complications were assessed. The distance between the distal end of the first-indwelled POD and the microcatheter tip was defined as the “landing distance,” and this was also evaluated via fluoroscopic analysis.

**Results:**

POD deployment was successful in 17 of 18 patients. The median landing distance was 9.6 mm, and no distal POD migration was observed after the formation of anchor loops.

**Conclusions:**

The specific anchoring function of the POD enables effective proximal occlusion of medium-sized vessels.

## Background

Proximal occlusion using metallic coils is sometimes technically challenging. A bigger coil in diameter relative to target vessel is difficult to form the anchor. On the other hand, a smaller coil relative to target vessel is possible to tightly compact, but it has high risk of migration. The penumbra occlusion device (POD; Penumbra Inc., Alameda, California, USA) is a unique hybrid coil, which has specific anchor function to achieve occlusion of medium-sized vessels. Specifically, the distal tip of the POD device is designed stiffer and larger in diameter than the rest of the device. Thus, the distal end of a POD can serve as an anchor, and the rest of the device can be securely stuffed into the anchor segment (Jambon et al. 2017; Petitpierre et al. 2016; Spiotta et al. 2016). Our aim was to retrospectively assess the safety and efficacy of embolization and to especially investigate the anchoring function of the POD for embolization of medium-sized vessels.

## Materials and methods

### Patients

The Institutional Review Board approved this retrospective study. We obtained patient information from our hospital database. Between September 2016 and November 2018, embolization using the POD was performed in 61 patients. Among these patients, cases using the POD for mere coil packing without anchoring function were excluded. A total of 18 patients (median age: 69 years; age range: 42–80; 11 men, 7 women) were selected for inclusion in the study. The characteristics of the patients are listed in Table 1.

**Table 1:** Patient characteristics and medical history

Case	Disease	Indication of embolization
1	IPMN	PAN after PpPD
2	Insulinoma	PAN after PpPD
3	PAVM	Occlusion of AV shunt
4	PAVM	Occlusion of AV shunt
5	Renal aneurysm	Renal aneurysmal rupture
6	Bile duct cancer	PAN after PpPD
7	Bile duct cancer	PAN after PpPD
8	Cholecystitis	PAN after cholecystectomy
9	IPMN	PAN after PpPD
10	Bile duct cancer	PAN after PpPD
11	Renal aneurysm	Renal aneurysmal rupture
12	Papilla vater cancer	PAN after PpPD
13	Bile duct cancer	PAN after PpPD
14	Bile duct cancer	PAN after HPD
15	PAVM	Occlusion of AV shunt
16	Pancreatitis	Splenic PAN
17	Bile duct cancer	PAN after PpPD
18	Hepatic aneurysm	Hepatic aneurysm

*IPMN* intraductal papillary mucinous neoplasm, *PAN* pseudoaneurysm, *PAVM* pulmonary arteriovenous malformation, *PpPD* pylorus preserving pancreatoduodenectomy, *AV shunt* arteriovenous shunt

### Interventional procedure

Under local anesthesia, a femoral puncture using the Seldinger technique and selective catheterization were performed in all patients. In the case of a pseudoaneurysm (PAN), parent arterial embolization was planned using the “isolation technique”; namely, the embolization of vessels both distal and proximal to the PAN was performed. The diameter of the target vessel was calculated from selective angiography. The size of a POD used was chosen based on the size of the target vessel. For diameter of a target vessel of 3.25–4 mm, 4 mm - 5 mm, 5 mm - 6 mm and 6 mm - 8 mm: POD 4, POD 5, POD 6, and POD 8 was chosen, respectively. The POD was delivered through a 0.025-in. microcatheter (ProgreatΩ, Terumo, Tokyo, Japan). Additional PODs or other metallic coils were used until hemostasis was obtained on a case-by-case basis.

### Measured outcomes

**Fig. 1:**
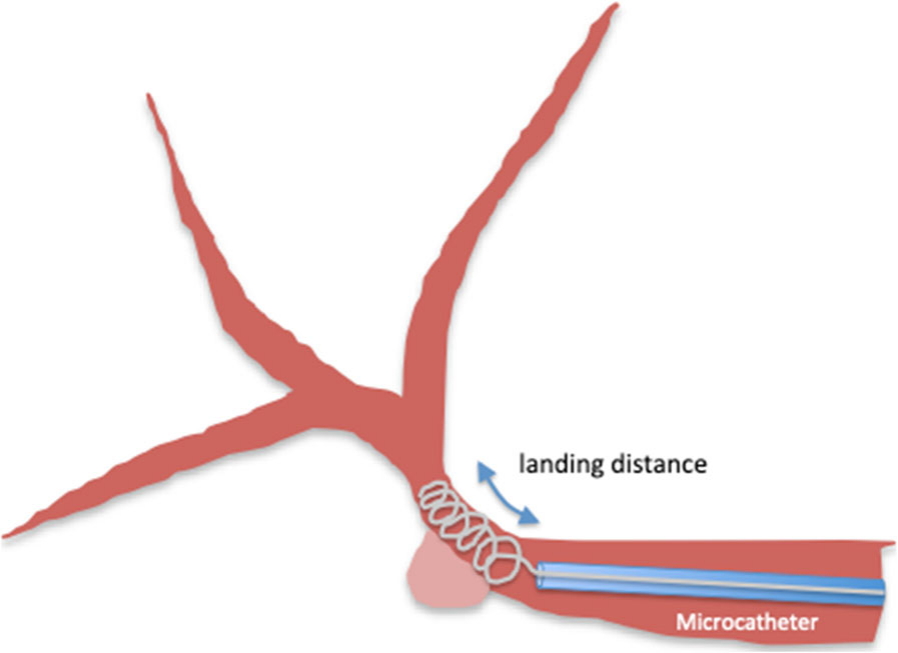
The distance between the distal end of the first penumbra occlusion device and the microcatheter tip was defined as the “landing distance,” and this was evaluated via fluoroscopic analysis

**Fig. 2:**
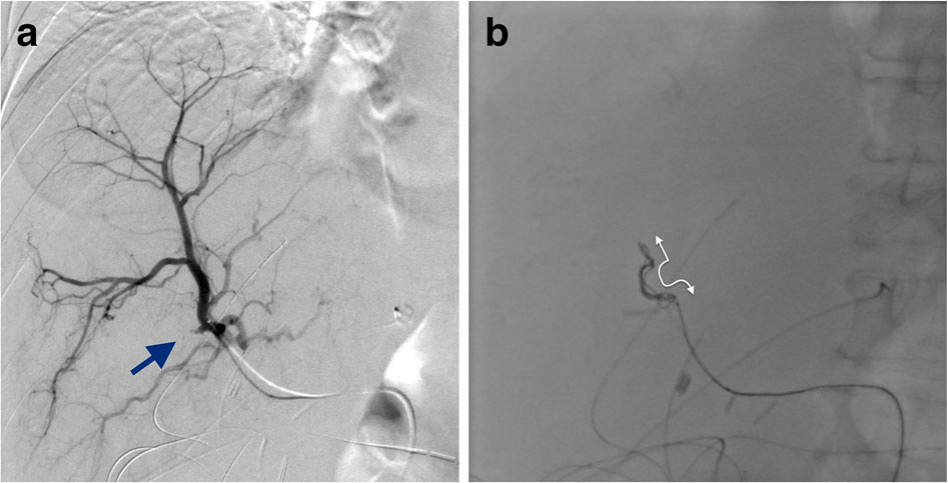
**a** Three weeks after pylorus preserving pancreatoduodenectomy, bleeding from the cholecystic arterial stump (arrow) occurred. **b** Embolization around the cholecystic arterial stump using a 4-mm penumbra occlusion device. The two-way arrow shows the landing distance

Technical outcomes and complications were assessed. The fluoroscopic movie, if available, was also reviewed to assess the anchoring function during the deployment of the first indwelling POD. A POD comprised an anchoring segment and a packing segment. During POD deployment, the anchoring segment began to stabilize the artery following the packing of the remnant coil. Thus, the distance between the distal end of the anchor segment and the microcatheter tip was defined as the “landing distance,” and this was evaluated via fluoroscopic analysis (Figs. 1 and 2).

## Results

**Fig. 3:**
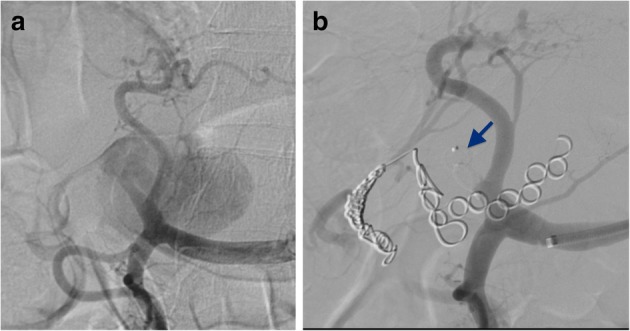
**a** A huge aneurysm of the right hepatic artery. **b** A vascular plug (arrow), instead of a penumbra occlusion device, was placed in the proximal right hepatic artery

Proximal arterial embolization using the anchoring function of the POD was performed in 18 patients. The target vessels in the 18 patients were as follows: proper hepatic artery (*n* = 9), right hepatic artery (RHA, *n* = 3), pulmonary artery (*n* = 3), renal artery (*n* = 2), and splenic artery (*n* = 1). POD deployment was successful in 17 of 18 patients. In a patient with a huge aneurysm of the RHA, the isolation technique was planned, and embolization of the distal RHA using the POD was successful. However, the POD could not be deployed in the proximal RHA because the proximal landing distance of the RHA was short enough to preserve left hepatic flow, and the POD could not be deployed in this patient. Instead of a POD, a vascular plug was placed in the proximal RHA (Fig. 3). Fluoroscopic analysis during deployment of the first indwelling POD was assessed in 15 patients. The target-vessel diameter in each patient was between 1.7 and 7.0 mm, and the optimal POD size was chosen in each case. The median landing distance was 9.6 mm (range 1.4–20.9) (Table 2). After the distal end of the POD formed anchor loops that adhered to the vessel wall, no distal migration was observed on fluoroscopic analysis.

**Table 2:** Endovascular outcomes

Case	Embolized artery	Diameter of target artery (mm)	First indwelling POD	Landing distance (mm)	Additional coils
1	Replaced RHA	4.7	6 mm × 50 cm	16.6	Ruby coil
2	RHA	3.3	4 mm × 30 cm	10.0	Ruby coil
3	PA	3.8	4 mm × 30 cm	6.3	Ruby coil
4	PA	2.9	4 mm × 30 cm	11.8	–
5	RA	4.3	4 mm × 30 cm	20.9	Ruby coil
6	CHA	3.5	4 mm × 30 cm	6.3	Ruby coil
7	CHA	7.0	8 mm × 60 cm	16.7	Ruby coil
8	RHA	4.1	4 mm × 30 cm	10.0	POD, Ruby coil
9	RHA	3.0	4 mm × 30 cm	^a^	Ruby coil
10	CHA	5.7	5 mm × 30 cm	^a^	POD, Ruby coil
11	RA	2.0	4 mm × 30 cm	20.2	POD, Ruby coil
12	CHA	1.7	4 mm × 30 cm	6.9	POD, Ruby coil
13	CHA	3.3	4 mm × 30 cm	9.6	Ruby coil
14	CHA	2.5	4 mm × 30 cm	8.1	POD, Ruby coil
15	PA	5.2	5 mm × 30 cm	1.4	POD
16	SA	4.7	4 mm × 30 cm	8.6	POD, Ruby coil
17	CHA	4.2	5 mm × 30 cm	8.6	POD, Ruby coil
18	RHA	5.2	5 mm × 30 cm	^b^	POD, AVP

*RHA* right hepatic artery, *CHA* common hepatic artery, *RA* renal artery, *PA* pulmonary artery, *AVP* Amplatzer vascular plug, *SA* splenic artery

^a^fluoroscopic movie was not available, and anchoring distance could not be verified

^b^POD could not be deployed due to unsuccessful anchoring

## Discussion

For embolization in cases, such as PAN and arteriovenous shunt, the proximal occlusion of target vessels is challenging. Special attention must be paid during the procedure to avoid the migration of the embolic device. The introduction of a POD is expected to solve this problem. The structure of the POD is unique with the distal end designed to work as an anchoring segment that enables proximal occlusion. Based on our fluoroscopic analysis, the median landing distance was 9.6 mm, and no POD migration was observed after the anchoring segment was successfully deployed. However, the deployment of the anchoring segment was unsuccessful in one case in our study. Presumably, an insufficient landing distance relative to the arterial flow caused the unsuccessful POD deployment. Thus, in this case, a vascular plug might be an alternative embolization technique. Stent grafting is also another technique for shielding a PAN (Sapna et al. 2011). Nevertheless, when the anchoring segment of the POD is successfully deployed, tight coil packing is possible. Moreover, the POD has specific advantages of navigation in tortuous vessels using a microcatheter (Jambon et al. 2018). Therefore, the POD seems to be an effective tool for the proximal occlusion of medium-sized vessels in various targets.

## Conclusions

Our fluoroscopic analysis indicates that the specific anchoring function of the POD enables effective proximal occlusion for medium-sized vessels in most cases. However, anchor formation is occasionally difficult in some situations, and other techniques are necessary for safer complete occlusion.

## Data Availability

Please contact author for data requests.

## Abbreviations

POD: penumbra occlusion device
PAN: pseudoaneurysm
RHA: right hepatic artery

## References

[CR1] Jambon E, Hocquelet A, Petitpierre F (2018). Proximal embolization of splenic artery in acute trauma: comparison between penumbra occlusion device versus coils or Amplatzer vascular plug. Diagn Interv Imaging.

[CR2] Jambon E, Petitpierre F, Brizzi V (2017). Proximal occlusion of medium-sized vessels with the penumbra occlusion device: a study of safety and efficacy. Cardiovasc Intervent Radiol.

[CR3] Petitpierre F, Lasserre AS, Tricaud E (2016). Proximal embolization of the splenic artery with a penumbra occlusion device (POD): a novel occlusion technique for blunt splenic injuries. Cardiovasc Intervent Radiol.

[CR4] Sapna P, Patel J, McPherson S (2011). Hemorrhagic complications after Whipple surgery: imaging and radiologic intervention. AJR.

[CR5] Spiotta AM, Turner RD, Chaudry MI (2016). Carotid sacrifice with a single penumbra occlusion device: a feasibility study in a swine model. J Neurointerv Surg.

